# Social Pension Scheme and Health Inequality: Evidence From China's New Rural Social Pension Scheme

**DOI:** 10.3389/fpubh.2021.837431

**Published:** 2022-02-07

**Authors:** Hui Yuan, Shuoqi Chen, Guochen Pan, Lingyun Zheng

**Affiliations:** ^1^Department of Insurance, School of Finance, Zhongnan University of Economics and Law, Wuhan, China; ^2^Department of Insurance and Actuarial Science, Economics and Management School, Wuhan University, Wuhan, China

**Keywords:** social pension schemes, rural residents, health inequality, regression discontinuity design, health concentration index

## Abstract

Health equality is an essential component of social justice, and the social policies should be as conducive to promoting health equality as possible. Based on the data from China, this article uses the regression discontinuity design method and the technique of decomposition of concentration index to examine whether the social pension schemes can significantly reduce health inequality among the residents, and tries to compute the contribution rate of pension benefit in alleviating the health inequality. Our results show that the pension benefit can improve the health level of the rural subscribers, especially for the low-income population. Implement of New Rural Pension Scheme contributes to reducing the health inequality among the rural elderly with contribution rate of 39.32%. Our results contain important policy implications.

## Introduction

Health is a fundamental human right, and the issue of health equality has attracted significant attention and extensive discussion worldwide (1, 2). Excessive health inequality will disrupt social harmony, cause a series of problems such as income inequality, and pose immense threat to labor quality and sustainable economic development as well (3). Promoting health equality is an issue which widely attracts policymakers and scholars' attention. China has experienced rapid progress in many fields in the past several decades, but sometimes the development was unbalanced and the inequality among residents was even enlarged during this process. Health inequality is one of such instances (4). In the *Outline of the Healthy China 2030 Plan*, the Chinese government proposed to promote the equity of basic public services of health maintenance, and gradually narrow the gap of health status among the residents nationwide.

Health status can be affected by various factors. Newman's health awareness theory proposes that awareness is vital for individual's health, strong health awareness will lead to better health status (5). Grossman's health production theory states that as depreciable human capital, an individual's health is affected by genetics, income, environment, behavior, medical treatment, and other factors (6). Most scholars agree that health level is usually closely associated with economic conditions. Compared with residents with higher economic and social status, low-income residents have apparent disadvantages in health maintenance, thus arises health inequality (7). Specifically, compared with the low-income residents, the high-income residents have good working environments and comfortable living conditions, and are less likely to suffer from health problems (8), the lifestyle choices they make are relatively better for health (9), and they have more advantages in the accessibility and utilization of medical services (10). To make matters even worse, there may be a feedback effect between the health gap and the income gap, i.e., individuals with better health are more likely to obtain higher incomes, and individuals with poor health are generally difficult to achieve average income (11, 12). The widened income gap further exacerbates health inequality (13).

The concentration curve and concentration index are used to measure health inequality by some researchers and the results show that almost all countries and regions have health inequality favoring high-income residents (14–16). Some scholars analyze the factors which cause health inequality and measure their contributions by decomposing the concentration index (17–19).

Many social security programs launched by the government are designed to provide welfare to all the residents and reduce inequality in many aspects, especially in health. Chinese government launched its social security programs, mainly composing of social medical insurance scheme and social pension scheme, many years ago. Social medical insurance scheme targets to improve the accessibility for medical treatment for the subscribers, and promote the subscriber's health status (20). However, current social medical insurance scheme in China adopts the “payment-before-reimbursement” principle, by which the insureds are required to pay the medical expenses in advance when seeking medical treatment, then a certain proportion of medical expenses are reimbursed after treatment. Large amount of prepayment become one of the reasons for restricting low-income groups from seeking proper medical treatment (21). This situation is confirmed in many literatures which find that though social medical insurance improves the health level of the subscribers, the degree of health inequality is unexpectedly enlarged (22, 23). Different from the social medical insurance scheme, social pension scheme provides a flow of income to the subscribers on a regular basis and strengthen their financial capability in their old age, which may help alleviate the budget constraints in utilizing the medical services for the elderly (24), or/and promote their overall living environments (25), or/and make balanced nutritional diet more affordable (26). As one of the main sources of income for the rural elderly, pension benefit may have a significant impact on their health level and health inequality across different populations. The social pension scheme does not significantly narrow the income gap among the elderly, but due to the high sensitivity of income for the low-income residents, the effect of pension on low-income residents' health status may be stronger than the high-income residents (27). Thus, social pension scheme may be able to play a role in narrowing the health gap between the elderly at different income levels. If this theory holds, the government should take this into account when designing the social pension scheme to achieve better social equality. Unfortunately, the existing research has not yet reached a definite conclusion on this issue. This article tries to verify if social pension scheme can effectively diminish the health inequality among the elderly population to add evidence to the related literature and provide policy implications for the government to design better social security programs and achieve better health equality.

Specifically, this article adopts data from a large database named the China Family Panel Studies (CFPS) and employ the regression discontinuity design method (RDD) and the concentration index (CI) decomposition technique as the main analysis tools to analyze the effect of social pension scheme in promoting health equality among the elderly. Our empirical results show that: (1) Pension benefit has positive effect on the average health level of the elderly, but this effect is heterogeneous across residents of different income, low-income residents benefit more from the pension in health maintenance; (2) Pension helps alleviated health inequality among the subscribers with a contribution rate of 39.32%.

Contributions of this article to the literature is three-fold: firstly, this article estimates the heterogeneous impacts of pension benefits on the health of the elderly, enriches the researches on performance of social pension scheme and contains importance for policymakers to improve the qualification and subsidy standards of social pension scheme (especially in developing countries or emerging economies); secondly, the social pension scheme in China is still in the progress of continuous improvement, results derived from this article can be incorporated into the future policies to achieve better social equity; thirdly, the regression discontinuity design (RDD) method is rarely employed in the related literature before, this method is close to random experiments in nature and contains excellent properties to alleviate the endogenous problem and identify causality, thus the validity and unbiasedness of the estimation results are ensured.

This article is organized as follows: section Data and Methodology introduces the data and methodology, section Empirical Results displays the empirical results, section Conclusion gives the conclusive remarks.

## Data and Methodology

### Source of Data

Social pension schemes in China have been fractional. In this research, one scheme, i.e., the New Rural Social Pension (NRSP) scheme which was established for the rural residents in 2009 is concentrated on. Even though the NRSP was incorporated into Urban Residents Social Pension (URSP) scheme in 2014, the samples are only selected from the original NRSP program in this article to make sure that all the samples come from the same background and the benefits of pension are comparable.^1^

This article employs data collected from a large-scale national survey, the China Family Panel Studies (CFPS). The CFPS survey was directed by the Institute of Social Science Survey of Peking University. The survey covers 16,000 households in 25 provinces in China. Information on health, medical care, insurance, and income are included which can be used to picture China's social development and residents' lives. After deleting data with missing information, 16,763 samples are included in our study. Data and documentation of CFPS are available at http://www.isss.pku.edu.cn/cfps.

### Variables

#### Dependent Variable

The dependent variable in our research is the health status of the elderly. Self-rated health score is usually used in the previous literature to represent the real health status of the elderly (28). In order to be more accurate, this article combines self-rated health score and rated-by-others health score to generate a comprehensive index to represent the real health status of the rural elderly. Self-rated health score is obtained by the answer to the question “How do you evaluate your health?”, the options “unhealthy,” “average,” “relatively healthy,” “healthy,” and “very healthy” are assigned with 1–5 points, respectively, rated-by-others health score is evaluated by the interviewers of the survey, and 1–7 scores represent the range from “very poor” to “very good,” respectively. Since the dimensions of these two variables are different, according to Ho (29), these two variables are standardized and then added together to obtain the comprehensive index.

#### Core Explanatory Variables

The core explanatory variable used in our research is whether the subscriber receives the pension benefit. The samples whose registration type is “agricultural household” are kept, while those of other registrations are deleted. Samples which receives pensions other than NRSP are also deleted to ensure that the health effect is caused by receiving the rural pension benefit.

#### Covariates

The following control variables are selected: demographic characteristics, including age, gender, and marriage; socioeconomic characteristics, including the type of job,^2^ enrollment in the NRSP, average household income, and quality of medical services (30, 31). Variable definitions and descriptive statistics are shown in Table 1.

**Table 1:** Descriptive statistics.

**Variables**	**Variable description**	**Mean**	**Variance**	**Min**	**Median**	**Max**
Health	The score of health scale	−0.95	1.738	−4.98	−1.15	2.67
Enrollment in NRSP	1 = yes, 0 = no	0.69	0.464	0	1	1
Age	Age of the respondent	68.06	6.052	60	67	95
Gender	1 = female, 0 = male	0.49	0.500	0	0	1
Marriage	1 = married, 0 = unmarried	0.81	0.394	0	1	1
Type of job	1 = agricultural labor, 0 = non-agricultural labor	0.68	0.465	0	1	1
Enrollment in NRCMS	1 = yes, 0 = no	0.90	0.294	0	1	1
Average household income	Logarithm of the average income of the household members	7.55	1.180	3.70	7.59	11.05
Quality of medical services	1 = very dissatisfied, 2 = dissatisfied, 3 = average, 4 = satisfied, 5 = very satisfied	2.49	0.879	1	2	5

It can be seen from Table 1 that the average and median health status of the elderly are <0, indicating that the health status of most elderly people is below the average level, and the gap of health exists. The coverage rate of China's social pension scheme reached 69%^3^ in 2018, which means that nearly 30% of the elderly were excluded from social pension scheme. The average amount of pension benefit received by the elderly was 7,411.11 yuan^4^ in 2017, accounting for 12.65% of the total household income. Due to low prices in rural areas and low income for rural families, the pension benefit is relatively large amount of income for the elderly (31).

### Methodology

#### Fuzzy Regression Discontinuity Design

Employing traditional OLS model in this research may be encountered with the following concerns: first, self-rated health scores and rated-by-others health scores are reported by the interviewees and interviewers, respectively, so measurement errors are inevitable; second, it is impossible to fully consider all the factors which affect the health and health inequality of the elderly, so omitted variable bias may exist; third, enrollment in NRSP is based on the principle of voluntary participation, rural residents with good health or high income are more inclined to be enrolled, while those with poor health or low income may be unwilling or unable to participate in the scheme, which will lead to sample selection bias. Without addressing these endogenous problems, the OLS estimation results will be biased and inaccurate.

**Figure 1:**
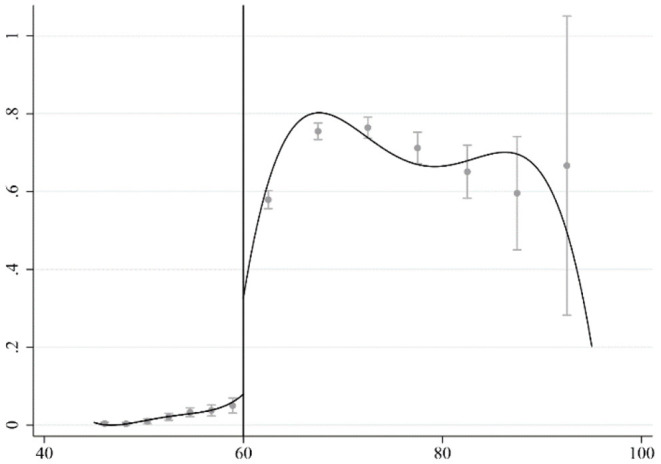
The probability of receiving pension benefit.

Figure 1 shows the probability of receiving pensions for the rural elderly. It can be observed that the probability of rural elderly obtaining pension benefit increases significantly at age of 60, as expected according to the policy provisions of NRSP. Interestingly, in practices, the social security agency generally conducts annual or quarterly review of pensioner eligibility, which means that an eligible elderly may not be able to receive pension at the inception of 60, but at some random time later, so we can only observe a jump in probability of receiving pension benefit (32, 33). Considering that, this article uses fuzzy regression discontinuity design (FRDD) to examine the causal effect of the NRSP pension benefit on the health of the rural elderly (34). Scholars generally believe that FRDD is closer to quasi-natural experiments, and the estimated results are more accurate. The formula used to calculate the probability is as follows:


(1) $$\begin{array}{l} \begin{array}{c} P\left(Pension_{i}=1|Age_{i}\right)=\{\begin{array}{cc} g_{1}(Age_{i}), & Age_{i}\geq 60\\ g_{0}(Age_{i}), & Age_{i}<60 \end{array} \end{array} \end{array}$$

Where *Pension*_*i*_ is the processing variable which indicates that the subscriber of NRSP receives the pension benefit, *Age*_*i*_ is the driving variable which represents the age of subscriber *i*.

The actual age of the rural elderly in the sample is continuous on both sides of age 60 when the subscriber is entitled to receive pension benefit according to the policy provisions. Falling on either side of the cutoff value is random without any human manipulation, thus it constitutes a quasi-natural experiment around the cutoff value. To obtain the local average treatment effect (LATE), which is the causal effect of the NRSP on the health of the rural elderly, we can choose an appropriate kernel function form and bandwidth, and perform least-squares estimation in the local neighborhood of the cutoff (35, 36), The formula used to calculate the LATE is as follows:


(2) $$\begin{array}{l} \begin{array}{c} \text{LATE}=\text{E}\left[\left({Health}_{i1}-{Health}_{i0}\right)|{Age}_{i}=60\right]\\ =\frac{limE_{\epsilon \to 0^{+}}\left({Health}_{i1}|{Age}_{i}=60+\varepsilon \right)-limE_{\epsilon \to 0^{-}}\left({Health}_{i1}|{Age}_{i}=60+\varepsilon \right)}{limE_{\epsilon \to 0^{+}}\left({Health}_{i1}|{Age}_{i}=60+\varepsilon \right)-limE_{\epsilon \to 0^{-}}\left({Pension}_{i1}|{Age}_{i}=60+\varepsilon \right)} \end{array} \end{array}$$

Where *Health*_*i*_ represents the health status of the elderly **i** in rural areas.

#### Measurement of Health Inequality and Decomposition of Contribution

Concentration curve and concentration index proposed by Wagstaff and Doorslaer (37) are employed to measure the health inequality of the elderly. The horizontal axis of the concentration curve represents the cumulative percentage of population income, and the vertical axis represents the cumulative percentage of population health. A concentration curve from the lower-left corner of the axis to the upper-right corner of the axis can be drawn. When the health indicator is a positive value, the concentration curve will be located below the diagonal if better health goes with people with higher level of incomes; otherwise, it will be located above the diagonal. The distance between the concentration curve and the diagonal line represents the degree of health inequality, and the farther the distance, the higher the degree of health inequality. Value of concentration index equals to twice the measure of area between the concentration curve and the diagonal. When the concentration curve is below the diagonal, it indicates that health inequality exists, and when it is above the diagonal, it indicates no health inequality. The greater the absolute value of the concentration index, the higher the degree of health inequality. We use the following formula to calculate the concentration index:


(3) $$\begin{array}{l} \begin{array}{c} \text{CI}\left(\text{Health}|\text{Income}\right)=\frac{1}{n}\overset{n}{\underset{i=1}{\sum }}\left[\frac{{Health}_{i}}{\bar{H}}\left(2R_{i}-1\right)\right] \end{array} \end{array}$$

Where $\bar{H}$ represents the sample mean of the variable health, and *R*_*i*_ represents the degree of deviation of individual's income ranking which can be obtained by sorting all samples from high to low according to income and calculate the degree of deviation of individual's ranking from the median.

In order to understand the contributions of each factors that affect health inequality, this article follows Wagstaff and Doorslaer (37) to decompose the health concentration index. According to the method, the health concentration index is decomposed as the product of the elasticity of health to affecting factors and the concentration index of those factors. The elasticity of health to affecting factors indicates the percentages of change in health caused by one percent change of the affecting factors, which represents the direct impact on health; the concentration index of the affecting factors represents the indirect impact of the affecting factors on health inequality. The decomposition formula is as follows:


(4) $$\begin{array}{l} \begin{array}{c} \text{CI}\left(\text{Health}|\text{Income}\right)=\underset{k}{\sum }e_{k}CI\left(X_{k}|Income\right)+\frac{CI_{\varepsilon }}{Ī}\\ =\underset{k}{\sum }\frac{\alpha_{k}{\bar{X}}_{k}}{\bar{H}}CI\left(X_{k}|Income\right)+\frac{CI_{\varepsilon }}{Ī} \end{array} \end{array}$$

Where *X*_*k*_ represents the factors which affect health inequality, *e*_*k*_ represents the elasticity of health to influencing factors, *CI*(*X*_*K*_|Income) represents the concentration index of affecting factors, *CI*_ε_ represents the concentration index of observably factors, Ī represents the sample mean of income, α_*K*_ represents the impact of affecting factors on health, and ${\bar{X}}_{k}$ represents the sample mean of affecting factors.

In order to decompose the health concentration index, an OLS model is run on the basis of propensity score matching (PSM) correction^5^:


(5) $$\begin{array}{l} \begin{array}{c} {Health}_{k}=\alpha_{0}+\alpha_{1}{Pension}_{k}+\underset{k=2}{\sum }\alpha_{k}X_{k}+\mu_{k} \end{array} \end{array}$$

Where μ_*k*_ represents the random disturbance term. Certain procedures are followed to obtain the contribution rate of affecting factors. Firstly, conduct a regression analysis to estimate the impact of each affecting factor on health α_*k*_; secondly, calculate the elasticity of health on each affecting factor *e*_*k*_ and the concentration index of each affecting factor CI(*X*_*K*_|Income); finally, use Equation (4) to obtain the contribution rate^6^ of each affecting factor on health inequality.

## Empirical Results

### Impact of NRSP on Health Status

#### Basic Regression Results

**Figure 2:**
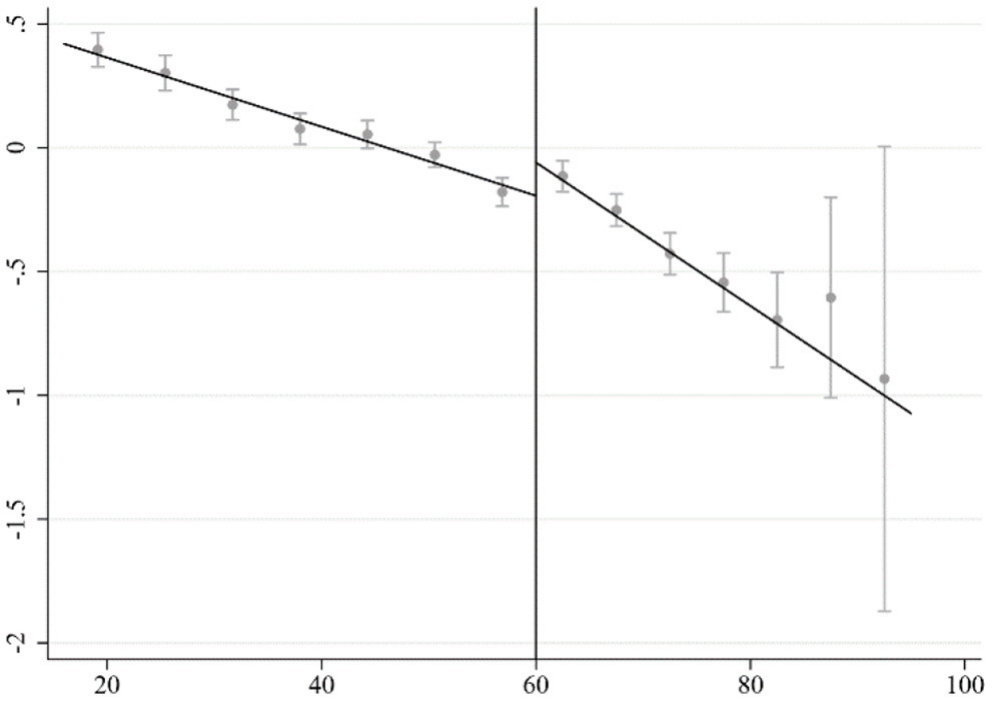
Distribution of health status of rural residents at different ages.

Figure 2 shows the distribution of the health status of rural residents of different ages. As can be seen from the figure, there is a significant jump in the health of the rural elderly around the cutoff value set by the regression discontinuity design model in this article. Some researchers argue that the health of the elderly could be improved due to reduced work pressure and increased leisure time after retirement (38). However, rural elderly people are mainly engaged in agricultural work, and there is no clear retirement time. They will continue to work until they are incapacitated (39). Receiving the pension benefit is the only identifiable factor to explain the difference (the jump). It is also worth noting that as the age increases, the health status of the elderly decline sharply. This is in line with common medical sense. The downward trend is more significant on the right side of the cutoff value, indicating that if traditional linear regression is used, the estimated result will be definitely biased. This also justifies the appropriateness of RDD method used in this article.

The estimated results of applying RDD are reported in Table 2. The requested bandwidth is calculated based on the IK method (40). Triangular kernel function is adopted as in most literature, and different forms of standard errors are used to cross check the robustness of results. Columns (1) and (2) report the non-parametric estimation results with and without control variables, respectively. The regression results show that the pension benefit significantly improves the health status of the rural elderly. This may be due to the function of pension benefit to meet the basic living needs of the rural elderly, as pension benefit can increase the income level and relax budget constraints.^7^ Pension benefit can also make balanced nutritious diets more affordable, improve the living environment, and increase the utilization of medical services, etc., thus contributes to the health status of the rural elderly. It is noted that the results of the regression discontinuity design could be quite sensitive to the choice of bandwidth. Different forms of kernel functions could sometimes change the estimation results dramatically (34). It is shown in the columns (3)–(6) that the results are still robust when altering age bandwidths and the kernel function forms.

**Table 2:** The impact of NRSP on health of rural elderly.

	**(1)**	**(2)**	**(3)**	**(4)**	**(5)**	**(6)**
Conventional	1.1310^**^	1.1745^**^	2.8890	0.7330^***^	1.0236^**^	0.9527^**^
	(0.4664)	(0.4647)	(2.3849)	(0.2037)	(0.4200)	(0.4516)
Bias-Corrected	1.3204^***^	1.3713^***^	3.4347	1.1003^***^	1.1849^***^	1.0612^**^
	(0.4664)	(0.4647)	(2.3849)	(0.2037)	(0.4200)	(0.4516)
Robust	1.3204^**^	1.3713^**^	3.4347^**^	1.1003^***^	1.1849^**^	1.0612^**^
	(0.5593)	(0.5604)	(1.6870)	(0.3198)	(0.5083)	(0.5299)
Bandwidth	Requested Bandwidth	Requested Bandwidth	Half Requested Bandwidth	Twice Requested Bandwidth	Requested Bandwidth	Requested Bandwidth
Kernel Type	Triangular	Triangular	Triangular	Triangular	Epanechnikov	Uniform.
Control Variables	NO	YES	YES	YES	YES	YES
Observations	10594	10210	10210	10210	10210	10210

*** *p < 0.01*,

** p < 0.05, *p < 0.1.

In order to avoid the selection bias when estimating the causal effects, validity tests of the cutoffs, the smoothness tests of the control variables, and the placebo tests are carried out. The validity tests of the cutoff identify that the individuals cannot enter the treatment group or control group by manipulating driving variables, and the distribution on both sides of the cutoffs is symmetric. The smoothness tests of the control variables confirm that the LATE is caused by receiving the pension benefit, but not the changes of other factors. The placebo tests in which the age cutoffs are changed show that the methods are sound and the results are robust.^8^

### Heterogeneous Effect of NRSP on Health Status of the Elderly

In order to verify the heterogeneous effect of the pension benefit on the health status of the rural elderly, the samples are divided into low-income, middle-income, and high-income groups according to the 30th and 70th percentiles of a family's per capita income. Table 3 reports the estimated results. The results show that the pension benefit significantly improves the health status of the elderly in low-income groups. The health effect on the elderly in the middle- and high-income group is positive, but not significant. Empirical results show that pension benefit will help alleviate income-based health inequality among the elderly.

**Table 3:** Heterogeneous effects of pension benefit on health status of the elderly.

	**(1)**	**(2)**	**(3)**
	**Low-income group**	**Middle-income group**	**High-income group**
Conventional	2.0040^*^	1.9641	1.3204
	(1.1886)	(2.4053)	(1.0802)
Bias-Corrected	2.6905^**^	2.3434	1.2493
	(1.1886)	(2.4053)	(1.0802)
Robust	2.6905^*^	2.3434	1.2493
	(1.4527)	(2.9477)	(1.2924)
Bandwidth	Requested bandwidth	Requested bandwidth	Requested bandwidth
Kernel type	Triangular	Triangular	Triangular
Control variables	YES	YES	YES
Observations	4,264	3,569	1,806

***p < 0.01,

** *p < 0.05*,

* *p < 0.1*.

### Impact of Pension Benefit on Health Inequality of the Elderly

#### Measurement of Health Inequality

**Figure 3:**
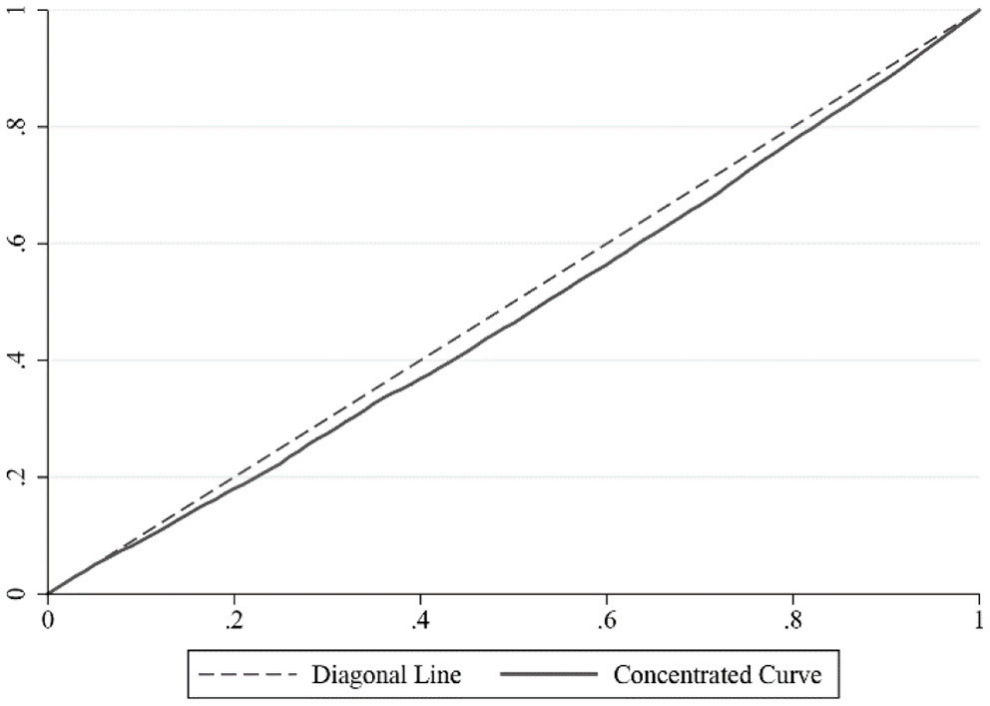
Measurement of health inequality among rural elderly.

Equation (3) is used to calculate the health concentration index of the full sample, and the result is 0.022. The concentration curve is located below the diagonal line (see Figure 3), indicating that there is real health inequality among the rural elderly population in China, the higher the income, the healthier the rural elderly residents.

#### Contribution of NRSP to Alleviating Health Inequality

Based on the regression results of the propensity score matching correction, the health concentration index is decomposed with Equation (4). Table 4 reports the elasticity, concentration index, and contribution rate of each affecting factor. The decomposition results show that the pension benefit alleviates the health inequality among the rural elderly with a contribution rate of 39.32%. The elasticity coefficient of health to pension benefit is positive, indicating that the NRSP has a direct positive impact on the health of the rural elderly, while the negativity of concentration index of the pension benefit indicates that there is no discrimination in the implementation of the policy, and the low-income elderly in rural areas can be subscribed into the NRPS as others. The combined effect of these two factors makes the pension benefit an important contributor to alleviating the health inequality among rural elderly residents.

**Table 4:** Decomposition of health inequality among rural elderly.

**Variables**	**Elasticity**	**Concentration index**	**Contribution (%)**
Enrollment in NRSP	1.7189	−0.0544	−39.32
Gender	0.3381	0.0083	1.18
Marriage	−1.0484	−0.0927	40.86
Type of job	−4.8782	−0.0070	14.25
Enrollment in NRCMS	−0.2043	−0.0315	2.71
Average household income	0.0941	0.0877	3.47
Medical level of medical treatment point	2.8963	0.0026	3.22

In addition to the pension benefit, type of job, marriage, enrollment in the NRCMS, average household income, and medical service quality are also important factors affecting health inequality. Type of job, marriage, and enrollment in the NRCMS are factors to exacerbate the health inequality. Specifically, engaging in agricultural work will worsen the health status of the rural elderly, thereby exacerbating health inequality; compared with the families with only one aged person, the families with two or more aged people may find it harder to maintain the old people's health due to limited resources; there is no inequality in the implementation of the NRCMS, and more preference is given to low-income rural elderly people. However, the “payment-before-reimbursement” principle is still a constraint for the low-income rural elderly people to enjoy the benefits of the NRCMS. Therefore, health inequality is inevitably exacerbated. Difference in average household income is the main cause of health inequality. Increase in household income can be helpful for improving the health of the rural elderly, but it can do little to alleviate the health inequality. The medical quality may positively affect the health status of the rural elderly, but the problems of poor access to medical resources and low frequency of medical services utilization for the low-income rural elderly have not been fundamentally solved, so the improvement of medical quality will probably benefit the high-income rural elderly more, exacerbating health inequality among the whole population.

## Conclusion

Employing the data from the China Family Panel Studies (CFPS), this article uses the regression discontinuity design method (RDD) and the concentration index (CI) decomposition technique to empirically study the impact of the NRSP on the health inequality of the elderly in China rural areas. The research results show that: First, health inequality is identified among the rural elderly in China; second, the impact of NRSP on health status of the rural elderly display heterogeneous, the health status of low-income elderly is found to be significantly improved due to pension benefit, while the impact is insignificant for the middle- and high-income elderly; third, by significantly improving the health of low-income rural elderly people, the NRSP narrows the health gap between the rural elderly of different income and reduces health inequality with a contribution rate of 39.32%. In conclusion, social pension schemes not only can improve the health status of the pensioners but also alleviate the health inequality among the elderly.

Our results contain meaningful implications for policy making: first, considering the positive impact of pension benefit on residents' health, government should endeavor to increase the level of pension benefits to achieve a better health result of the residents; second, government should tries to improve the enrollment rate of the residents by providing stronger financial subsidies toward the low-income family, these family will benefit more in terms of health; third, government should care more about the vulnerable population with “health-poverty” by providing free pension benefits to the elderly who are extremely poor, or disabled, or without dependant.

Nevertheless, this article has certain limitations. First of all, it is difficult to measure the health status of the people accurately, we considered both self-rated health score and rated-by-others health score to reflect the health status, but this measurement may be still subject to subjective bias. Secondly, fuzzy regression discontinuity design helps to control observable and unobservable factors, with cross-section data in this article, there may still be confounding effects caused by unobservable time-varying factors. Thirdly, the technique of decomposition of concentration index is useful, but the theories used to explain the results are not rich enough. For future researches, we believe it is important to extend the study to the whole population since the social pension schemes are becoming integrated, and the mechanism through which the pension scheme affects the health inequality also needs to be clarified.

## Data Availability Statement

Publicly available datasets were analyzed in this study. The data can be found at: http://www.isss.pku.edu.cn/cfps.

## Author Contributions

HY contributed to conception and design of the study. SC processes the data and writes the early draft of the article. GP helps in developing the research idea and contributes some intellectual contents to the draft. LZ helps in modeling and drafting. All authors contributed to manuscript revision, and approved the submitted version.

## Conflict of Interest

The authors declare that the research was conducted in the absence of any commercial or financial relationships that could be construed as a potential conflict of interest.

## Publisher's Note

All claims expressed in this article are solely those of the authors and do not necessarily represent those of their affiliated organizations, or those of the publisher, the editors and the reviewers. Any product that may be evaluated in this article, or claim that may be made by its manufacturer, is not guaranteed or endorsed by the publisher.

## Footnotes

^1^Therefore, we still use the name of New Rural Social Pension (NRSP) in the following discussions.

^2^Agricultural labor is defined as work in agriculture, forestry, animal husbandry, sideline, and fishery, and non-agricultural labor is defined as work in other industries.

^3^Data source: Calculated by the data released at the regular press conference of the Ministry of human resources and social security in the second quarter of 2021.

^4^Data source: Calculated by the data of CFPS2018.

^5^The decomposition of the concentration index requires regression coefficients, while the regression discontinuity design method adopts nonparametric estimation. Therefore, this part uses the OLS model for estimation and the PSM method to alleviate the endogenous problem. When estimating the propensity score function, the logit model and kernel matching method are selected.

^6^A positive contribution rate means exacerbating the health inequality, and a negative value means alleviating the health inequality.

^7^Although the NRSP squeezed out the transfer payments of children, relatives, and friends to a certain extent, the income of the rural elderly increased on the whole (46).

^8^Due to space limitation, the robustness test results are not reported. Interested readers can contact the author.
